# Programmed genome rearrangements in ciliates

**DOI:** 10.1007/s00018-020-03555-2

**Published:** 2020-05-27

**Authors:** Iwona Rzeszutek, Xyrus X. Maurer-Alcalá, Mariusz Nowacki

**Affiliations:** 1 Institute of Biology and Biotechnology, Department of Biotechnology, University of Rzeszow, Pigonia 1, 35-310 Rzeszow, Poland; 2 Institute of Cell Biology, University of Bern, Baltzerstrasse 4, 3012 Bern, Switzerland

**Keywords:** Ciliates, Nuclear dimorphism, Genome rearrangement, DNA elimination, Small non-coding RNAs

## Abstract

Ciliates are a highly divergent group of unicellular eukaryotes with separate somatic and germline genomes found in distinct dimorphic nuclei. This characteristic feature is tightly linked to extremely laborious developmentally regulated genome rearrangements in the development of a new somatic genome/nuclei following sex. The transformation from germline to soma genome involves massive DNA elimination mediated by non-coding RNAs, chromosome fragmentation, as well as DNA amplification. In this review, we discuss the similarities and differences in the genome reorganization processes of the model ciliates *Paramecium* and *Tetrahymena* (class Oligohymenophorea), and the distantly related *Euplotes*, *Stylonychia*, and *Oxytricha* (class Spirotrichea).

## Introduction

Developmentally regulated genome rearrangements (DRGRs) involve the elimination of specific DNA sequences (from the germline) somatic cell lineages. In most cases, this phenomenon is associated with two forms of DNA elimination either: (a) chromosome elimination where the entire chromosome is lost [1] or (b) chromosome diminution, a process characterized by loss of chromosome portions through chromosome breakage and repair during the developmental transformation from germline to soma [2, 3].

Programmed DNA elimination was first described in 1887 by Theodor Boveri [4] in the horse parasitic nematode, *Parascaris univalens*. Since then, DRGRs have been identified in diverse multicellular organisms including nematodes, arthropods, hagfish, lampreys [3] and lymphoid lineages of vertebrates [5]. However, it appears most pervasive in ciliates, an ancient clade of microbial eukaryotes (> 1 Gya; [6]), where genome rearrangements lead to the elimination of 30–95% of the germline genome [7–9]. This review will focus on genome rearrangements in the two best-studied classes of ciliates: the Oligohymenophorea (including *Paramecium* and *Tetrahymena*)*,* and members of the Spirotrichea (including *Euplotes*, *Oxytricha and Stylonychia*).

## Ciliates as a model organism

Ciliates are unicellular eukaryotes found in diverse environments (fresh/saltwater as well as soil) across the globe that emerged more than 1 billion years ago [6]. Due to their morphological and morphogenetic characters, the taxonomy of ciliates has been ambiguous for a long time. Numerous studies have improved the phylogenetic relationship between ciliates with the rest of the eukaryotic tree of life, being members of the Alveolata (along with apicomplexans and dinoflagellates) [10]. Similarly, phylogenomic studies within Ciliophora illustrate the great diversity and deep evolutionary history despite limited taxon sampling [11]. Even though just a handful of the ~ 4500 described ciliate species have been studied in-depth [12], they share complex cytoskeletal structures, well-developed ciliary structures at the cell surface (for swimming, food uptake and sensing environmental signals), the separation of germline and somatic genomes into distinct nuclei (nuclear dimorphism), as well as DRGRs (reviewed in [13]). Although the majority of studies are limited to the model genera *Tetrahymena*, *Paramecium*, and *Oxytricha*, these models have greatly contributed to our understanding of biological mechanisms and phenomena present in diverse eukaryotic lineages. This includes discovery and description of ribozymes [14], the discovery of the first histone-modifying enzyme [15], variant nuclear genetic codes [16–20], the initial identification of telomerase and telomere structure [21, 22], numerous examples of small RNA-mediated heterochromatin formation [23, 24], as well as mechanisms enabling the transcription of short DNA fragments [25].

## Nuclear dimorphism in ciliates

**Fig. 1:**
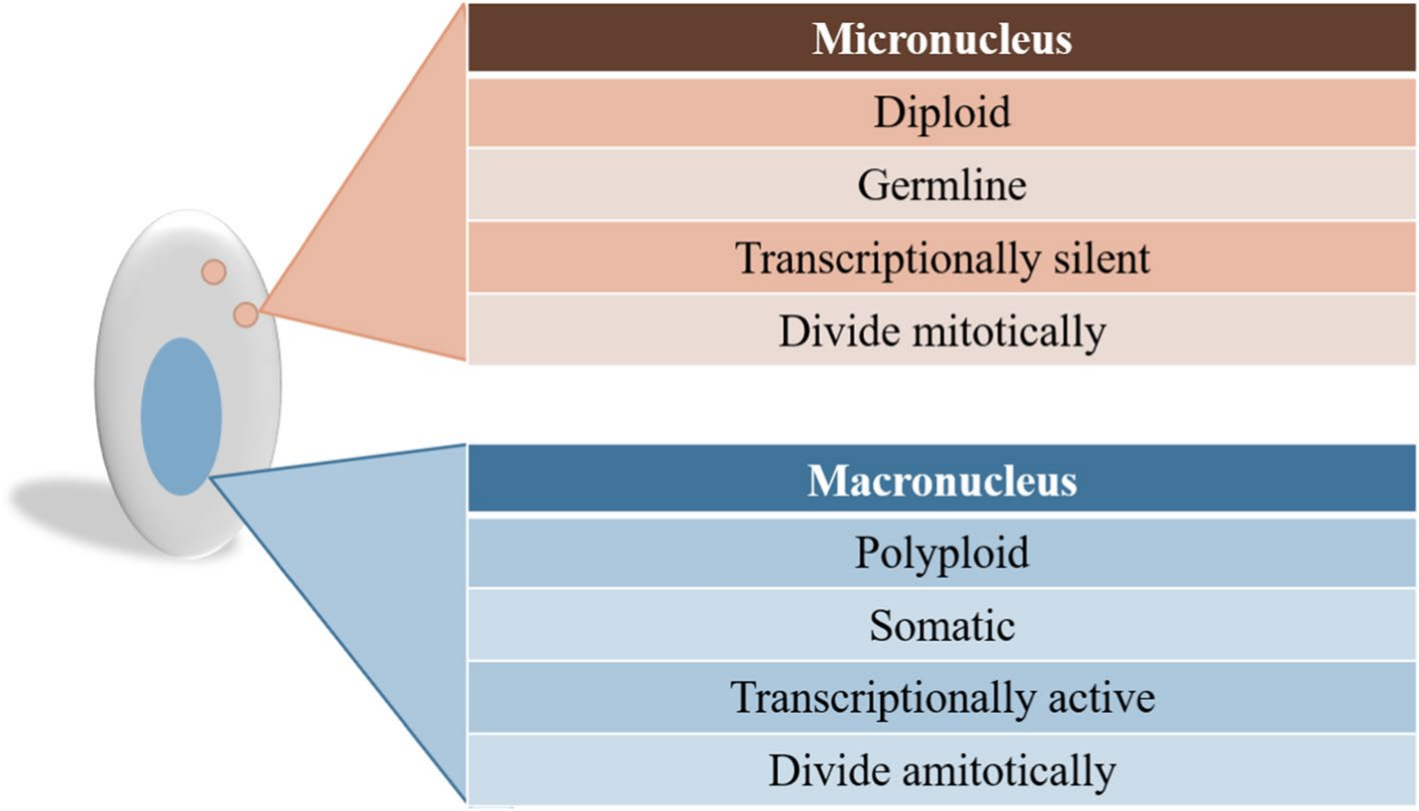
Representative differences between germline and somatic nuclei ciliates

In multicellular eukaryotes, germline and somatic functions are separated into distinct cell types, (e.g. pollen versus leaf in plants, spore versus hyphae in fungi, or egg versus skin in humans). However, in ciliates, both germline and somatic genomes co-exist within a single cell, providing each cell with at least one somatic nucleus used for gene expression and one germline to propagate the genome across sexual generations (Fig. 1).

Each ciliate cell possesses at least one micronucleus (MIC) and one macronucleus (MAC); however, their number varies between the species (reviewed in [26]). Interestingly, all micronuclei present in the cell possess features of typical eukaryotic nuclei, (i.e. diploid [27–29], centromeres [30] and are transposon rich [30, 31]). The MAC is transcriptionally active throughout the entire life cycle and possesses highly processed chromosomes. These MAC chromosomes are gene-rich, lack centromeres and can range in ploidy from ~ 2 N in the Karyorelictea to > 13,000 N in the Heterotrichea [32–34]. During asexual growth, the hyper-polyploid MACs divide amitotically, which lacks mitotic spindles and chromatin condensation, separating chromosomes in bulk as large masses, which can result in daughter nuclei with unequal amounts of DNA. The degree of inequality in the segregation of DNA to the two MACs during amitosis can be exacerbated under environmental stressors [35, 36]. Moreover, MAC chromosomes are amplified to elevated copy numbers [26]. For instance, in Oligohymenophorea, each MAC chromosome is present in the equal copy number, namely,  ~ 45 copies of each 225 chromosomes in *T. thermophila* [37, 38] and ~ 800 copies of each chromosome in *P. tetraurelia* [39, 40]*.* In contrast, the *Oxytricha* MAC harbours thousands of unique gene-sized nanochromosomes, which are amplified to ~ 1900 copies [31]. However, unlike *Paramecium* and *Tetrahymena*, these nanochromosomes are maintained at unique copy numbers, varying between a few hundred to 10^6^ copies [26, 41–43].

**Fig. 2:**
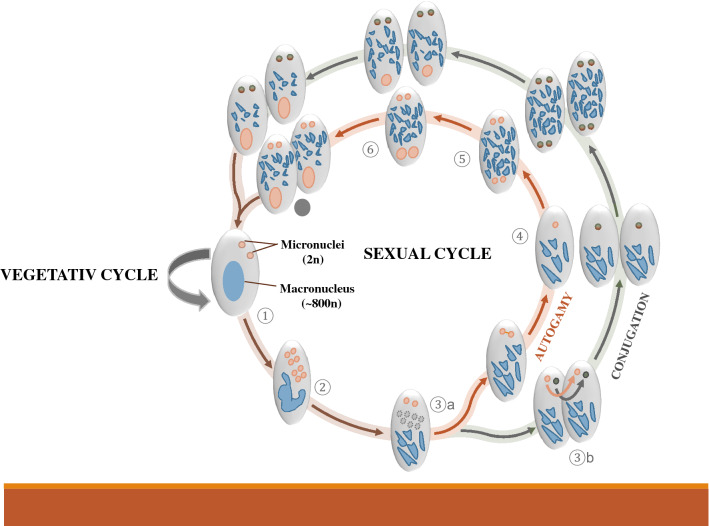
The *Paramecium* sexual cycle (autogamy and conjugation). (1) A vegetative cell with diploid micronuclei and polyploid macronucleus. (2) Meiosis of the micronuclei and beginning of old macronucleus fragmentation. (3a) Mitotic division of one remaining micronucleus (seven out of eight micronuclei degenerates) leading to the production of two identical gametic nuclei. (3b) Alternatively, during conjugation, exchange of haploid nuclei occurs. (4) Zygotic nuclei formation through the fusion of two haploid products. (5) Two subsequent mitotic divisions of the zygotic nuclei. (6) Differentiation of the two mitotic products into new macronuclei. (7) Caryonidal division/separation leading to the formation of two cells each containing two micronuclei and new macronucleus as well as fragments of old macronucleus

During vegetative (or asexual) growth, the germline remains transcriptionally inactive and divides mitotically [44]. This changes during sex or self-fertilisation/autogamy (Fig. 2) [45]. At the onset of sex and development, micronuclei undergo meiosis and are fused with a partner haploid MIC that gives rise to the zygotic nucleus from which new micro- and macronuclei are formed.

### Features and origin of internal eliminated sequences

The discovery that germline-limited internal eliminated sequences (IESs) resemble transposon sequences brought a new challenge for scientists trying to understand the mechanisms underlying their elimination. Jacobs and Klobutcher [46] observed that IESs in *Euplotes crassus* possess the consensus sequence 5′-TATrGCRN-3′ (Y = pyrimidine, R = purine), which resembles terminal inverted repeats (TIRs) at the end of their Tec family transposable elements [46–48] and *Paramecium*’s Tc1/Mariner transposons [46–49]. All IESs found to date in *Euplotes and Paramecium* possess 5′-TA-3′ dinucleotide repeats at each boundary, where a single copy of the dinucleotide remains in the new macronucleus after excision. Based on these observations, Klobutcher and Herrick [47, 50] developed a model for the origin of IESs, where transposons initially invade the germline genome, then spread throughout, and ultimately decay over time into the identifiable IESs currently found in ciliate germline genomes.

These sequences must be excised during the development of a MAC to produce a functional somatic genome. IESs are present in all the ciliate germline genomes studied to date [30, 31, 48, 51–53], although in varying amounts (~ 12,000 IESs in *Tetrahymena thermophila* [54], ~ 45,000 IES in *Paramecium tetraurelia* [48, 51] and > 200,000 IESs in *Oxytricha trifallax* [31]*.* IESs are typically AT rich (70–100%) and bounded by pairs of short direct repeats (most are 1-8 bp) that help identify the boundaries between Macronuclear Destined Sequences (MDSs) and IESs [30, 31, 48, 52]. Recently, Maurer-Alcalá et al*.* [53] demonstrated that MDS–IES boundaries are identifiable by sharp changes in GC content. For instance, in *P. tetraurelia* where IES excision is precise, GC content was decreased in close proximity to its MDS–IES boundaries. On the other hand, in *Tetrahymena* where almost all IESs are excised imprecisely, GC contents are characterized by the great variability associated with MDS–IES boundaries within the inferred MDS itself [53]. The length and genomic distribution of IESs in germline genomes are very diverse, with most IESs in *Tetrahymena* being intergenic and “long” (> 100 bp to over 10 Kbp) [55, 56], whereas the IESs in *Paramecium* and *Oxytricha* often interrupt protein-coding sequences and are comparatively short (most < 100 bp) [31, 48].

As IESs often disrupt coding regions for most ciliates, they must be accurately excised during development to enable expression of the functional genes in the newly developed MAC [48, 57–59]. However, in *Tetrahymena thermophila* nearly all of the 7350 well-described IESs (of the ~ 12,000 total IESs) are excised imprecisely, due to variable MDS–IES junction sites. The potential deleterious impacts of this imprecision are likely mitigated by the genomic distribution of these IESs, which are predominantly found in intergenic (6182; 82%) or intronic (1168; 16%) regions [30, 60–62]. In contrast, only the excision of transposon-like sequences and minisatellites in *Paramecium* is imprecise [63], whereas the ~ 45,000 IESs nestled within or near protein-coding sequences are precisely excised during macronuclear development, although small numbers of IESs are excised at alternative MDS–IES boundaries [40, 48, 64]. Overall, imprecise elimination results either in the fragmentation of micronuclear chromosomes into shorter acentromeric macronuclear chromosomes to which telomeric repeats are added, or to the imprecise re-joining of flanking sequences [63].

Previous work in *Paramecium* and *Tetrahymena* have shown that the pointer sequences present at both ends of IESs, influences the efficacy of IES excision. Analyses of *Paramecium’s* IESs have demonstrated that single basepair-mutations in the conserved terminal repeat of IESs lead to their retention during development [65–67]. Additionally, for some *Paramecium* IESs, flanking sequences are necessary for excision [68]. For example, the removal of a portion of the 72 bp flanking region of one end of a small 28 bp IES in *Paramecium* reduced the efficiency of excision, and complete removal of all wild-type sequences adjacent to the TA abolished excision [68]. Recently, it has been shown a small subset of *Paramecium’s* IESs shares a common 5 bp motif that is implicated in their sRNA-independent excision [69]. In *Tetrahymena,* flanking sequences are known to have a significant role in the elimination of a number of IESs [70, 71]. Together, Lia3p and Lia3-Like 1 (LTL1) regulatory proteins interact with flanking regulatory sequences to determine MDS–IES boundaries for several IESs for excision [72–74]. These data highlight the importance of both IES pointer sequences and their flanking regions in identifying MDS–IES boundaries.

Another variable that influences IES excision/recognition in *Paramecium* is its length. Swart et al. [75] indicated that the frequencies of IES sub-terminal bases change with IES length. Moreover, it has been shown that small IESs (shorter than 150 bp) are less sensitive to sRNAs depletion [76], suggesting that some IESs are more difficult to recognize/excise and require additional information (from the sRNAs) for their accurate excision.

## Developmentally regulated genome rearrangements

**Fig. 3:**
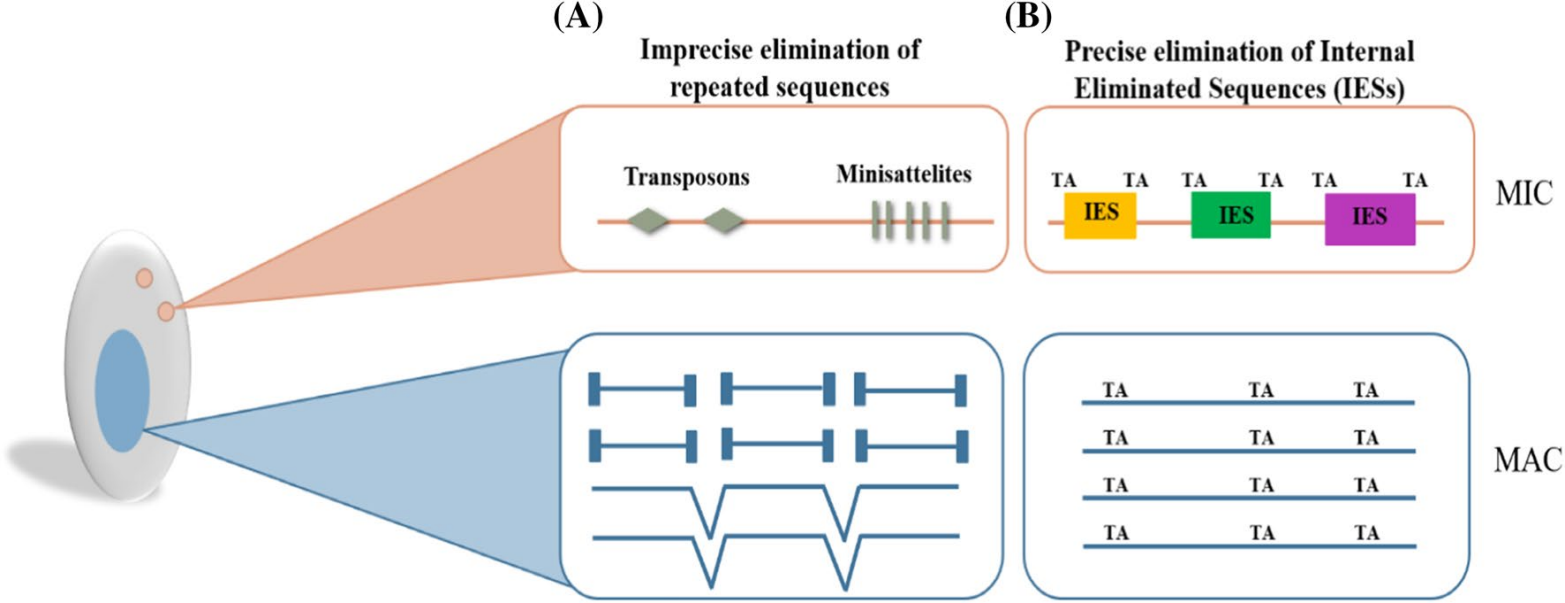
Macronuclear differentiation process is shown on *Paramecium* example. **a** Imprecise elimination of repeated sequences like minisatellites and transposons followed by re-joining of the flanking sequences or de novo telomere addition. **b** Precise excision of internal eliminated sequences (IESs) possessing two TA repeats at each boundary one copy of which remains after excision

Although the mechanistic details behind ciliate DRGRs differ between even closely related taxa (*i.e. P. tetraurelia* and *T. thermophila*), the basic principles of this phenomenon are conserved (Table 1). In the developing macronucleus, rapid DNA synthesis takes place and interstitial DNA sequences such as transposons, minisatellites and IESs are excised. Afterward, the hundreds to thousands of broken chromosome ends created during excision are rejoined through non-homologous end-joining mechanisms [77–79], followed by de novo telomere addition, and finally chromosome amplification (Fig. 3)*.* The end result is the production of a new functional somatic nucleus that contains the streamlined transcriptionally active chromosomes that maintain cell. This section is devoted to describing these phenomena in more detail.

**Table 1:** Differences between the genome reorganization processes among ciliates

	*Paramecium tetraurelia*	*Tetrahymena thermophila*	*Oxytricha trifallax*
MIC chromosomes	?	5 [37]	?
MAC chromosomes	188 [39, 40]	225 [38]	15,600 [43]
Nanochromosomes	No	No	Yes [43]
Unscrambling	No	No^a^	Yes [178]
IES percentage	30% [48]	30% [179]	90% [31]
IES location	Genic and intergenic regions [48]	Intergenic Regions [90]	Genic and intergenic regions [180]
Small RNA source	MDS and IES [25, 81]	Biased towards IES [111]	MDS [83]
Small RNA target	IES [81]	IES [80]	MDS [82, 83]
DNA methylation	6 mA (*P. aurelia*) [132]	6 mA [131]	5mC, 6 mA [127, 135]

^a^One locus showing unscrambling [181]

### Role of small noncoding RNA in programmed genome rearrangements

An important breakthrough in our understanding of the regulation of DNA elimination was unravelling the involvement of noncoding RNA (ncRNA) in this complex epigenetic process. Briefly, the MIC is bi-directionally transcribed producing long transcripts that are processed into small RNAs. In *Paramecium* and *Tetrahymena* (cl: Oligophymenophorea), these sRNAs then “scan” the parental MAC. Those sRNAs corresponding to the parental MAC are lost, leading to the enrichment of MIC-matching sRNAs [23, 76, 80, 81]. However, in *Oxytricha* and *Stylonychia* these sRNAs are produced from transcripts derived from the parental MAC genome and are putatively involved in protecting MDSs in the MIC rather than identifying IESs for elimination (as in *Paramecium* and *Tetrahymena*) [82–84]. This reflects the extreme differences in germline genome content as ~ 30% of the germline genome is eliminated in Oligohymenophorean ciliates versus ≥ 95% of the germline in spirotrichs. These sRNAs pools ultimately help delineate MDSs and IESs, although details differ among even “closely” related species (*e.g. Paramecium* and *Tetrahymena*).

### Small RNA-mediated programmed genome rearrangements in Oligohymenophorea

**Fig. 4:**
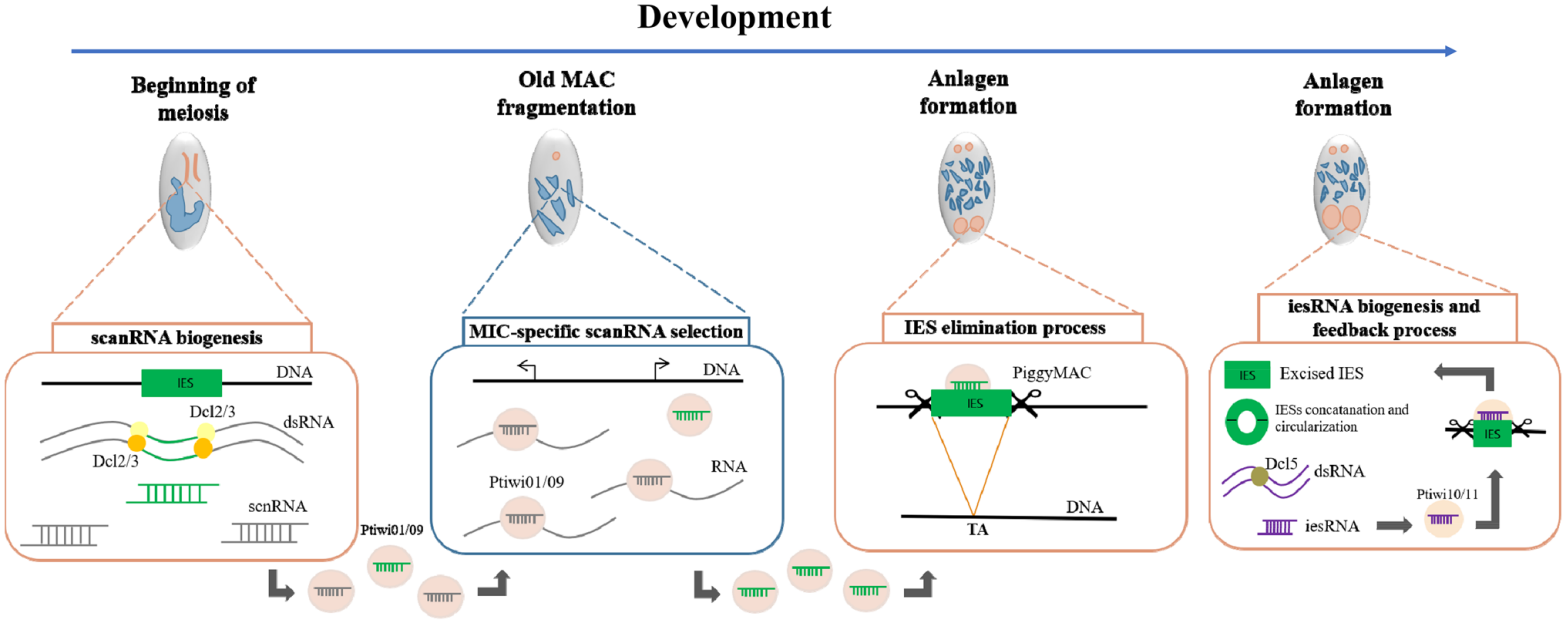
The scanning model in *Paramecium*. **a** Bi-directional transcription of the parental micronuclear genome during meiosis and scnRNA production by Dcl2/Dcl3. **b** Scanning process between scnRNA and transcript of somatic DNA. **c** scnRNA targeting IES for excision by PiggyMac. **d** Concatenation and circularization of excised IESs. Transcription of excised and circularized IESs to produce dsRNA. dsRNA cleavage by Dcl5 to produce iesRNA ensuring elimination of all copies of IESs. Old macronucleus degradation and development of the new macronucleus

The first insights that DNA elimination relies on homologous RNA molecules originated from work in Oligohymenophorean ciliates (i.e. *Paramecium* and *Tetrahymena*). During prophase of meiosis, the MIC is bi-directionally transcribed [85] by RNA polymerase II [86], generating large MIC-based transcripts. These transcripts are then processed into small “scan” RNAs (scnRNAs are 25 nt in *Paramecium* and 25–29 nt in *Tetrahymena*), by Dicer-like ribonucleases (Dcl1 in *Tetrahymena* and Dcl2 and Dcl3 in *Paramecium*), that “scan” for homologous sequences in the parental MAC genome (Fig. 4a) [76, 81, 87–89]. Initial experiments suggested that scnRNA production from MIC-derived transcripts, represented relatively equal representation of IES and MDS regions [23]. However, recent work in *Tetrahymena* shows two “pulses” of sRNA production, which are associated with enriched transcription and processing of IES sequences [90]. Interestingly, the biased production of *Tetrahymena* scnRNAs is predominantly from type-A IESs (per-centromeric and telomeric regions of the MIC chromosomes) [91].

After production in the micronuclei, scnRNA duplexes are transported to the cytoplasm where they are loaded onto the PIWI family proteins, (Twi1p in *Tetrahymena* [23, 80] or Ptiwi1/9 complex present in *Paramecium* [92, 93]) which are then transported to the parental macronucleus (Fig. 4b). There, the genome “scanning” effectively removes the MAC-matching sRNAs, enriching for micronuclear-limited scnRNAs from the initial population. These MIC-enriched scnRNAs are transported to the developing macronucleus to guide DNA excision (Fig. 4c). Interestingly, it has been suggested that *Paramecium’s* scnRNAs bind to longer RNA transcripts, rather than directly to DNA, in both the old and new MAC [94, 95]. In both *Tetrahymena* and *Paramecium*, there is a second wave of sRNAs that aid in ensuring the accurate identification and excision of IESs [76, 91]. In *Tetrahymena,* these “late scnRNAs” are produced from both types of IESs [Type-A and Type-B (located at the chromosomal arms)] *in cis*. These late scnRNAs are loaded onto the Twi1p and Twi11p complexes, which further guide heterochromatin formation *in trans* and ensure the elimination of all IESs copies [91]. However, in *Paramecium*, excised IESs are eventually circularized, with smaller IESs concatenated together prior to circularization [25]. These IES concatemers act as the transcriptional template for iesRNAs, which further ensure the precise and accurate excision of IESs [25]. These secondary iesRNAs are produced after IES excision in *Paramecium*, whereas *Tetrahymena*’s “late” scnRNAs are produced prior to any IES excision [96]. Compared to *Tetrahymena* where early and late scnRNAs are produced by the same Dicer-like ribonuclease (Dcl1), *Paramecium* iesRNAs are produced by Dcl5 [76]. As in *Tetrahymena* the primary scnRNAs and secondary iesRNAs are associated with distinct Piwi proteins, Ptiwi01/09 with scnRNAs and Ptiwi10/11 carries the iesRNAs [93]. Together, the primary and secondary sRNAs ensure the faithful elimination of all copies of IESs present in the developing MAC genome leading to the production of a new functional macronucleus (Fig. 4d).

### Small RNA-mediated programmed genome rearrangements in Spirotrichea

DNA elimination in *Oxytricha* (Spirotrichea) is quite distinct from the distantly related Oligohymenophorea. While *Paramecium* and *Tetrahymena* generate scnRNA in the parental MIC, *Oxytricha’s*, 27-nt-long small RNAs derive from the transcription of the parental macronucleus rather than the germline [82, 83]. In addition to that, these 27mers have been shown to associate with PIWI homologs called Otiwi1 (hence called PIWI-interacting RNAs, piRNAs). The injection of 27 nt piRNAs corresponding to IESs into developing *Oxytricha* leads to the retention of those IES in the new somatic genome [83]. These data, combined with the apparent parental MAC origin of the piRNAs, suggest that they are responsible for identifying macronuclear destined sequences (MDS) to protect against excision, rather than targeting IESs for excision, as in *Tetrahymena* and *Paramecium*. Interestingly, as in *Paramecium*, *Oxytricha* also circularizes some excised TE and non-repetitive germline-limited sequences that are also actively transcribed [97, 98]. This presence of development-specific extrachromosomal circular DNA was originally described in *Euplotes* (cl: Spirotrichea) [99, 100], although the circularization process and content appears to differ between *Oxytricha* and *Paramecium*. If these circularized products of excised IES and transposon-like Tec elements in *Euplotes* lead to the production of small RNAs remains undetermined. Despite the differences in sRNA sources and targets, ciliates have evolved a relatively efficient and low-energy cost means to distinguish soma and germline.

### DNA unscrambling

**Fig. 5:**
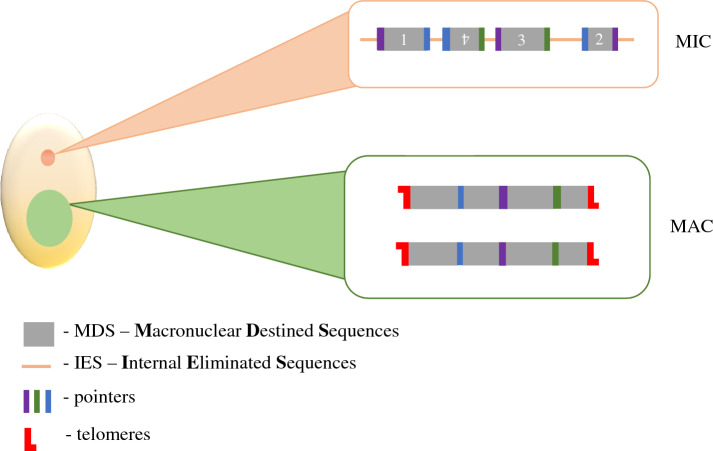
Unscrambling process in *Oxytricha*

In addition to delineating somatic and germline-limited DNA, macronuclear development in *Oxytricha* requires a very spectacular form of DNA rearrangement called unscrambling [26, 31, 101]. In the germline, MDSs can also be disordered and/or found on both strands of DNA (*i.e.* “inverted”) and may even originate from distant germline loci (Fig. 5) [31]. An extreme example is *DNA polymerase* α in *O. nova*, *O. trifallax* [102, 103] and *S. lemnae* [104], which is broken into more than 40 MDS present at two distinct loci separated by > 3 kbp. Work in *Oxytricha* has demonstrated that DNA unscrambling is directed by long RNA templates derived from the parental macronucleus [105–107]. These long template RNAs, in conjunction with unique pointer sequences, act as a reference aiding in the accurate reordering of MDSs [108, 109]. The accuracy of DNA unscrambling is incredibly sensitive to these template RNAs. For example, microinjection of alternately unscrambled templates (e.g. swapping the order of MDSs) leads to the production of macronuclear chromosome resembling the introduced template [105]. Furthermore, RNAi knockdown of these long RNA templates results in aberrant or reduced rearrangements of MDSs in the resulting chromosomes found in the new MAC [105].

### Histone modification in DNA elimination

As in other eukaryotes, histone modifications play an integral role in the effective silencing of transposable elements and germline-limited DNA. In *Tetrahymena,* heterochromatin-specific marks, H3K9me3 and H3K27me3 are present in the MAC or both MIC and MAC, respectively [24, 87, 110–112]. Accumulation of H3K9me3 and H3K27me3 in the MAC is catalysed by histone methyltransferase Ezl1p, whereas Ezl2p is responsible for H3K27me3 in the MIC [111, 113]. As in other eukaryotes, small RNAs are involved in guiding the deposition of these conserved marks [110]. After deposition, specific marks are subsequently recognized by chromodomain-containing effectors. In particular, *Tetrahymana’s* Pdd1p (a homolog of HP1) accumulates on IESs, binding to methylated histones [114, 115] and is proposed to aid in recruiting *Tetrahymana’s* domesticated PiggyBac transposase (Tpb2p) for their excision [116]. Additionally, recent data indicated that RNAi-dependent *Polycomb* repression pathway is important for controlling transposable elements in *Tetrahymena* [117]. Disruption of the *Polycomb* repression pathway (knockout of DCL1, EZL1 and PDD1) results in the activation of TE transcription as well as the germline mobilization of TE [87, 110]. Moreover, numerous other histone modifications in *Tetrahymena* have been identified and may play roles in its DRGR [118, 119]. As in *Tetrahymena*, histone-specific marks such as H3K27 and H3K9 trimethylation are mediated by Ezl1 in *Paramecium* [120] and associated with chromatin assembly factor 1 subunit C-like protein (PtCAF-1) [121]. However, the developmental roles of these marks remain unclear.

As in Oligohymenophorea, heterochromatinization has been observed in Spirotrichea. During development, germline chromosomes are polytenized, with large blocks of observable heterochromatin prior to DNA elimination and fragmentation into thousands of unique gene-sized nanochromosomes [122]. In *Stylonychia*, this process is linked to the differential expression of a suite of histone H3 variants and subsequent post-translational modifications (PTM) [123–125]. For instance, H3K27me3 was shown to accumulate at the MIC-specific sequences prior to excision [126], while H3.7, acetylated at lysine-32, specifically associate with MDSs [123]. Moreover, knockdown of Piwi impacts the expression of histone H3.3 during macronuclear development in *Stylonychia*, implicating that H3.3 incorporation into nucleosomes is ncRNA-dependent [123]. Unfortunately, little is known about the roles of histone modifications and variants among other spirotrich ciliates, in *Oxytricha* and *Euplotes*.

### DNA modification in IES elimination

Besides marking IESs for elimination through histone modifications, chemical modifications of germline DNA may also play a role in ciliate DRGRs. 5-Methylcytosine (5mC) has been identified on some germline limited sequences (*e.g.* transposons and satellite repeats) in *Stylonychia* and *Oxytricha* [127, 128] as well as in aberrant DNA rearrangements and parental DNA undergoing degradation [127]. Additionally, azacytidine and decitabine (DNA methyltransferase-inhibiting drugs) induce demethylation of both somatic and germline DNA during DRGRs, further implicating 5mC as a specific marker for DNA elimination/degradation [127]. Moreover, 5mC in *Stylonychia* correlates with gene activity as well as with chromatin structure during macronuclear differentiation [129]. However, recent work in *Paramecium* was unable to detect any evidence for 5mC modifications, suggesting that these modifications may only be involved in the DRGRs of spirotrich ciliates [130].

While the function of 5mC DNA modifications and their phylogenetic distribution in ciliates remains unclear, the only widely conserved DNA modification is 6*N*-methyladenine (6 mA) [131–135]. Data from *Tetrahymena* shows that 6 mA is only present in the transcriptionally active MAC and is preferentially enriched in the consensus sequence 5′-AT-3′ [133, 134]. 6 mA modifications also localize to linker DNA regions downstream of the transcription start site (TSS of Polymerase II transcribed genes) and directly influence nucleosome positioning [133, 136–138]. *Tetrahymena*’s MT-A70 homologue ATM1 (6mA DNA methyltransferase) is required for the normal growth and development of the cell following sex [139]. Interestingly, the enzyme MTA1c (DNA methyltransferase) responsible for 6mA modifications in *Oxytricha* disfavours nucleosome occupancy, contrary to *Tetrahymena* [135]. Beh et al. [135] suggest that decreased nucleosome occupancy is due to dA:dT base pair destabilisation by 6mA, which decreases the DNA melting temperature. However, the exact mechanism remains undetermined.

Unfortunately, while DNA modifications during MAC development are present, exactly how they might direct IES excision and/or MDS protection requires further investigation.

### Transposases required for DNA excision

Transposase domestication has occurred throughout the eukaryotic tree of life, and can be linked to important DRGRs, such as those in ciliates and those involved in V(D)J recombination in animals [140, 141]. In *Paramecium* and *Tetrahymena*, IES excision is performed by a domesticated PiggyBac (PB) transposase (PiggyMac (Pgm) and TPB2 in *Paramecium and Tetrahymena*, respectively) [142, 143]. Excision of IESs in *Paramecium* by Pgm generates a 4 bp overhang at 5′ends centred around the "TA" dinucleotide pointer sequence showing the same geometry as those catalyzed in vitro by PB transposases [142, 144]. While Pgm is believed to carry out the physical excision, *Paramecium* possesses five accessory Pgm-like domesticated transposases (PgmL1-PgmL5) that interact with Pgm individually, with PgmL1 and PgmL3 directly involved in Pgm’s ability for precise IES excision [145]. Compared to *Paramecium’s* PiggyBac transposase, that possesses a sequence specificity (5′-TTAA-3′) [142], Tpb2p in *Tetrahymena* possess less stringent sequence specificity, as most of the IESs it excises are not flanked by any common motif [50, 62]. In *Tetrahymena* excision of the > 10,000 Tpb2p-dependent IESs is imprecise [116, 143]. *Tetrahymena* also possesses multiple transposase proteins, such as Lia5p (domesticated PB transposase) which localizes on IESs and facilitates Tpb2p-dependent IES elimination [114, 146] and is required for chromosome fragmentation in *Tetrahymena* [146]. In contrast to the most abundant IESs in *Tetrahymena*, the excision of 12 particular IESs (possessing TE features, such as terminal inverted repeats and the 5′-TTAA-3′ cutting site) is precise and depends on Tpb1p and Tpb6p [147, 148].

In *Oxytricha*, IES excision is triggered by telomere-bearing element (TBE) family transposases [149], which belong to a superfamily of transposase genes that possess a common DDE catalytic motif [150]. Analyses of the *Oxytricha* MIC genome found that complete and partial copies of *Tc1/Mariner* transposons constitute around 13% of the germline genome [151]. Compared to the *Paramecium’s* and *Tetrahymena’s* transposase-related proteins, TBEs in *Oxytricha* are encoded in the MIC genome itself, rather than the somatic genome [152], and cut with a 3 nt 5′ overhang at an ANT recognition site [98]. Similar to members of the Oligohymenophorea, *Oxytricha* possesses multiple transposases, all of which have a necessary role in its development [149].

### Chromosome fragmentation

Chromosome fragmentation is one of the major events that occur during macronuclear development in ciliates. The most extreme chromosome fragmentation takes place in ciliates with gene-sized chromosomes (e.g. *Euplotes*, *Stylonychia,* and *Oxytricha*) where their MIC chromosomes are fragmented into > 15,000 unique MAC chromosomes [43, 153]. Given the incredibly short size of these nanochromosomes (averaging ~ 2.8–3.2 Kbp in *Oxytricha and Stylonychia* [43, 153]), most (~ 90%) encode just a single open reading frame (ORF) [43, 154]. While less dramatic, the five MIC chromosomes present in *T. thermophila* are fragmented into ~ 225 multigene MAC chromosomes (from < 100–1500 Kbp) [155], whereas in *P. primaurelia,* MIC chromosome fragmentation gives rise to 50–1000 Kbp MAC chromosomes [156].

For some ciliates, the fragmentation of the MIC genome does not occur randomly, but at specific chromosome breakage sequences (CBS) in the germline [157]. In *Euplotes crassus*, a conserved 10 bp consensus sequence (*Euplotes*-chromosome breakage sequence; E-CBS: 5′-HATTGAAaHH’, H = A, C or T) directs a staggered double-strand break (DSB) at a precise distance and orientation, which provides the substrates for telomere addition [158–160]. In *Tetrahymena*, a conserved 15 bp chromosome breakage sequence (CBS: 5′-WAAACCAACCYCNHW-3′, W = A/T; Y = T/C; H = A/T/C; N = G/A/T/C) is necessary for chromosome fragmentation and telomere addition [30, 161–164]. While the E-CBSs in *Euplotes* are ultimately retained in the MAC [158–160], in *Tetrahymena* the CBSs themselves are germline-limited and are eliminated with 4–34 bp of flanking DNA on both sides [165]. Interestingly, in *Tetrahymena*, fragmentation of the germline at the CBSs generates 33 non-maintained chromosomes (NMCs) [30, 164, 166, 167]. Unlike the typical “large” (> 100 Kbp) somatic chromosomes in the MAC, these NMCs are generally short, ranging from 30 to 80 Kbp and have a limited life-span, either being degraded prior to de novo telomere addition or lost by ~ 120 asexual divisions [166]. While a majority of the NMCs harbour functional ORFs (some of which are actively transcribed) [30], it remains unclear what their role, if any, might be in the post-sexual life cycle or the transition from sexual immaturity to maturity.

### De novo telomere addition

The presence of DNA double-strand breaks (DSBs) is mostly known to be associated with the induction of repair machinery [Non-Homologous End Joining (NHEJ) or Homologous Recombination (HR)]. The relatively large number of DSBs associated with chromosome fragmentation during development in ciliates generates a large number of chromosomes whose broken ends are “healed” through de novo telomere addition. As in other eukaryotes, ciliate telomeres consist of tandem repeats at the 5′and 3′ends of their chromosomes, such as 5′-GGGGTTTT-3′ (G_4_T_4_) in *Oxytricha* and *Euplotes* [168], 5′-GGGGTT-3′ (G_4_T_2_) in *Tetrahymena* [22] and 5′-GGGGTT-3′ or 5′-GGGTTT-3′ in *Paramecium* [169, 170].

Although chromosome fragmentation is a very reproducible and relatively precise event, de novo telomere addition does not typically occur at precise nucleotide position (the exception being *E. crassus* [158]), generating micro-heterogeneity among the amplified chromosome copies in the developing macronucleus. In *Oxytricha* and *Tetrahymena*, telomere addition sites have been found to be clustered within regions ≤ 30 bp, [165, 171–173], whereas this is often ~ 1 to 2 Kbp in *Paramecium* [169, 170]. However, in *E. crassus*, there is no heterogeneity and telomeres are added at the same nucleotide positions in all macronuclear copies [158]. Additional heterogeneity can arise from the use of alternative chromosome fragmentation sites in *Paramecium* and *Oxytricha*. In *Paramecium,* the ends of some MAC chromosomes can be generated at alternative telomere addition sites separated by 2–13 Kbp. Each of the regions shows heterogeneity in the telomere’s positions [170, 174, 175]. While most of the chromosome fragmentation in *Oxytricha* results in gene-sized chromosomes, the use of alternative fragmentation sites (or failure to fragment) can result in macronuclear chromosomes encoding additional ORFs [154, 172, 173].

The exact mechanism of de novo telomere addition remains poorly understood. Data performed on *Tetrahymena* suggested the involvement of the telomere end binding homologue Pot2p in de novo telomere addition that exclusively localizes to CBSs during chromosome fragmentation [176]. Recent work from *Stylonychia* has shown that microinjection of RNA templates carrying variable telomeric repeats into the developing macronucleus leads to modified telomeres in vegetative cells suggesting that de novo telomere addition depends on a telomere-containing transcript derived from the parental macronucleus [177]. However, to understand this process in more detail, further work needs to be done.

## Conclusions

Ciliates are a diverse group of organisms that have deeply contributed to our recent knowledge about the regulatory role of epigenetics in development. Identification of sRNA pathways as well as histone modifications that mediate DNA elimination is providing a greater understanding of the genome reorganization process in ciliates while shedding new insight into the evolution of epigenetic processes across eukaryotes. While we have a basic understanding of the overall genome reorganization process, numerous outstanding questions remain open. How are IES regions identified and preferentially transcribed to produce sRNAs in the meiotic MIC? What, if any, role is there for retaining IESs in the MIC? How has this process evolved across the ciliate phylogeny? Further comparative analyses of somatic and germline genomes, and the associated DNA elimination process, will be instrumental in answering these questions and will ultimately shed light on the variety of RNA-mediated epigenetic pathways and the dynamic regulation of genome function and structure.
